# Cytokine Networks and Heart Failure Outcomes: CA125 as a Bridge Between Congestion and Inflammation

**DOI:** 10.3390/ijms26199527

**Published:** 2025-09-29

**Authors:** Enrique Santas, Arancha Martí-Martínez, Sandra Villar, Rafael de la Espriella, Enrique Rodriguez-Borja, Elena Revuelta-López, Arantxa González-Miqueo, Antoni Bayés-Genís, Juan Sanchis, Julio Núñez

**Affiliations:** 1 Cardiology Department, Hospital Clínico Universitario de Valencia, Instituto de Investigación Sanitaria (INCLIVA), 46010 Valencia, Spain; ensantas@gmail.com (E.S.); sanvc28@gmail.com (S.V.); delaespriella_raf@gva.es (R.d.l.E.); sanchis_juafor@gva.es (J.S.); 2 Faculty of Medicine and Dentistry, Universitat de València, 46010 Valencia, Spain; 3 Clinical Biochemistry Department, Hospital Clinico Universitario de Valencia, 46010 Valencia, Spain; aranchamartimartinez@gmail.com (A.M.-M.); rodriguez_enr@gva.es (E.R.-B.); 4 Centro de Investigación Biomédica en Red Enfermedades Cardiovasculares (CIBERCV), 28029 Madrid, Spain; erevuelta@igtp.cat (E.R.-L.); amiqueo@unav.es (A.G.-M.); abayesgenis@gmail.com (A.B.-G.); 5 Cardiology Department, Hospital Universitari Germans Trias i Pujol, Universitat Autònoma de Barcelona, 08916 Badalona, Spain; 6 Program of Cardiovascular Disease, CIMA Universidad de Navarra, Department of Cardiology and Cardiac Surgery, Clínica Universidad de Navarra and IdiSNA, 31008 Pamplona, Spain

**Keywords:** heart failure, inflammation, antigen carbohydrate 125, cytokines

## Abstract

Inflammation and congestion constitute fundamental mechanisms underlying heart failure (HF). Carbohydrate Antigen 125 (CA125) is a well-established biomarker in HF, primarily associated with congestion, but also it may act as a functional ligand amplifying the inflammatory response in HF. Our aim was to evaluate the potential modulatory effect of CA125 on inflammation, assessed by a set of cytokines (interleukin [IL]-6, IL-10, IL-1β, and tumor necrosis factor [TNF]). We prospectively included 284 patients admitted for acute HF in which cytokines and CA125 were assessed at admission. Study endpoints were all-cause mortality and total HF rehospitalizations. At a median follow-up of 4.2 years (interquartile range: 1.3–7.5), a total of 211 patients (74.3%) died, and 117 patients (41.2%) experienced 249 HF readmissions. In the multivariable analysis, a significant interaction between IL-6 and IL-10 and CA125 was observed for both outcomes (*p*-value for interactions < 0.05 for all comparisons). Among patients with CA125 > 35 U/mL, both IL-6 and IL-10 showed a positive, linear relationship with the risk of death or HF readmissions. In contrast, we did not find a significant association in patients with CA125 ≤ 35 U/mL. In conclusion, the association between IL-6 and IL-10 with long-term adverse events was significantly modulated by CA125 status, being significantly associated with poor prognosis only when CA125 was upregulated. These findings support a potential modulatory role for CA125 in the inflammatory response in HF.

## 1. Introduction

Inflammation plays a key role in the pathophysiology of heart failure (HF) [1,2]. Systemic inflammatory activation promotes HF progression by driving detrimental structural changes in the heart, impairing endothelial function, increasing oxidative stress, and contributing to volume overload [1,2,3,4,5]. The interleukin-1 (IL-1)-interleukin-6 (IL-6) signaling axis, together with broader cytokine-mediated pathways, has been linked to greater HF severity and adverse outcomes [6,7,8,9,10,11,12,13]. This inflammatory response is particularly pronounced during episodes of worsening HF and states of volume overload [3,9,10].

Carbohydrate antigen 125 (CA125), also known as mucin 16 (MUC16), has emerged as a valuable biomarker in HF, showing a strong association with interstitial and third space fluid accumulation, as well as with adverse clinical outcomes [14,15].

Structurally, CA125 possesses a heavily glycosylated extracellular domain capable of interacting with immune-related proteins [16]. CA125 expression by mesothelial cells is enhanced by inflammatory mediators such as tumor necrosis factor (TNF), interleukin-1β (IL-1β), and IL-6, underscoring its potential role in modulating immune pathways in HF [17], and suggesting that CA125 serves not merely as a biomarker but also plays a ligand-like role [18,19]. In line with this, the prognostic impact of other biomarkers linked to inflammation and fibrosis, such as galectin-3 (Gal-3) and soluble ST2 (sST2), appears to be accentuated in patients with higher circulating CA125 levels [20,21]. Recent studies also show CA125 expression in epicardial adipose tissue, colocalizing with inflammatory infiltrates and fibrosis markers, including TGF-β and collagen I [19]. These findings support the hypothesis that CA125 may actively participate in local inflammatory and fibrotic processes in HF and reinforce the interplay between inflammation and congestion. However, the potential interaction between CA125 and key pro-inflammatory cytokines in HF have not been previously evaluated.

In the current study we aimed to investigate whether CA125 status modifies the relationship between a set of key cytokines in HF (IL-1β, IL-6, IL-10, and TNF) and long-term adverse outcomes in patients with acute HF.

## 2. Results

The mean age of the cohort was 72.8 ± 11.4 years, 140 (49.3%) were women, and 157 (55.3%) had left ventricular ejection fraction (LVEF) ≥ 50%. Median (P25–P75) concentrations of cytokines were IL-6 = 13.8 pg/mL (5.8–42.0); IL-10 = 20.2 pg/mL (7.1–78.6); IL-1β = 0.4 pg/mL (0.1–2.4); and TNF = 7.6 pg/mL (3.7–19.9). Median CA125 was 69 U/mL (30–141) and the proportion of patients with CA125 > 35 U/mL was 68.3%. Baseline characteristics, overall and stratified by CA125 status, are summarized in Table 1 and Table A1. Compared with patients with CA125 ≤ 35 U/mL, those with higher CA125 levels presented with lower admission blood pressure, a greater prevalence of clinical signs of volume overload (e.g., peripheral edema, pleural effusion), and higher NT-proBNP concentrations. They also showed lower left and right ventricular systolic function. Cytokine concentrations did not differ significantly between CA125 groups.

### 2.1. Study Outcomes

At a median follow-up of 4.2 years (interquartile range: 1.3–7.5), a total of 211 patients (74.3%) died, and 117 patients (41.2%) experienced 249 HF readmissions. The annualized rates of all-cause mortality and HF rehospitalization were 17.7 and 28.8 events per 100 person-years, respectively. The distribution of HF readmissions per patient was as follows: 61 (21.5%) had one readmission, 22 (7.7%) had two, 15 (5.3%) had three, and 19 (6.7%) experienced four or more readmissions.

### 2.2. Cytokine Network and Risk of Adverse Clinical Events in the Whole Sample

**Figure 1:**
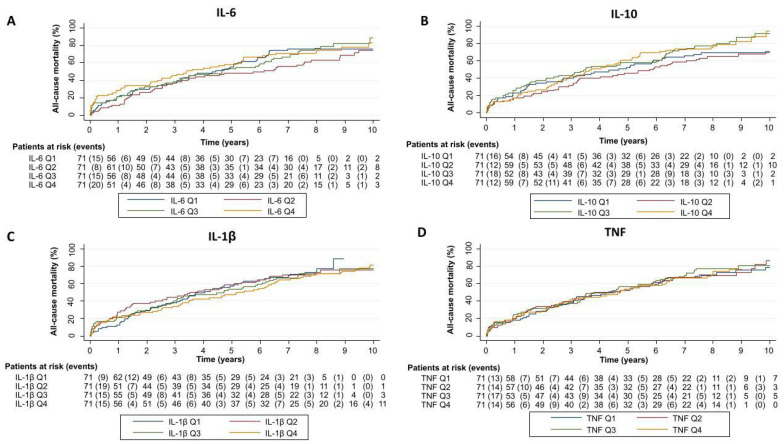
Kaplan–Meier curves for mortality across quartiles of cytokines. Cumulative all-cause mortality over 10 years is shown for patients stratified by quartiles (Q1–Q4) of (**A**) Interleukin-6, (**B**) Interleukin-10, (**C**) Interleukin-1β, and (**D**) Tumor necrosis factor. Patients at risk and number of events for each time point are displayed below each panel. IL: interleukin, TNF: tumor necrosis factor.

In the whole sample, we did not observe significant differences in the incidence of all-cause mortality across quartiles of IL-6, IL-1β, or TNF levels (Figure 1). Regarding HF readmissions, only patients in the two upper quartiles of IL-10 exhibited increased readmission rates over time (Table 2). After multivariate adjustment, none of the cytokines analyzed (included as main terms) were independently associated with an increased risk of all-cause mortality and total HF readmissions (Table 3).

### 2.3. The Modifying Prognostic Role of Cytokine Network Across CA125: Long-Term Mortality

**Figure 2:**
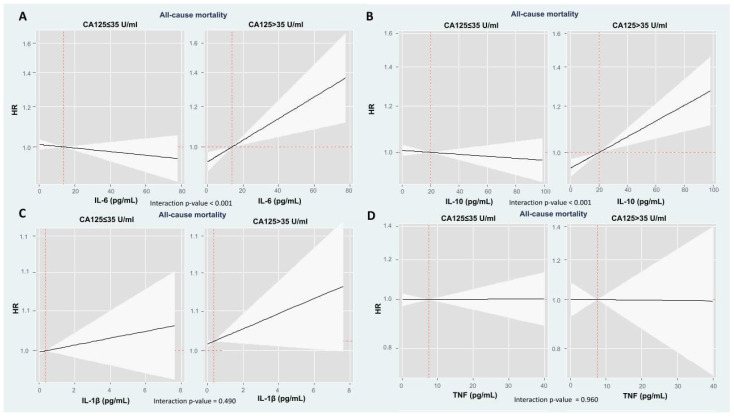
Hazard ratio depiction for mortality risk along the continuum of cytokines across CA125 status (CA125 ≤ 35 or >35 U/mL) in the multivariable model. Associations of IL-6 and IL-10 with mortality risk were modified by CA125 status, showing a positive relationship in patients with CA125 > 35 U/mL, but no significant association in those with CA125 ≤ 35 U/mL. IL-1β and TNF associations with mortality were not affected by CA125 levels. Solid black lines represent HR estimates, with shaded areas indicating 95% confidence intervals. The *p* for interaction tests whether the prognostic effect of cytokines on mortality risk differs according to CA125 levels. (**A**) Interleukin-6: *p* for interaction < 0.001; (**B**) Interleukin-10: *p* for interaction < 0.001; (**C**) Interleukin-1β: *p* for interaction = 0.490; and (**D**) Tumor necrosis factor-α: *p* for interaction = 0.960. CA125: antigen carbohydrate 125, HR: hazard ratio, IL: interleukin, TNF: tumor necrosis factor.

Multivariate analyses revealed that the associations of IL-6 and IL-10 with mortality risk were significantly modified by CA125 status (*p*-values for interactions < 0.001). Among patients with CA125 > 35 U/mL, both IL-6 and IL-10 showed a positive, linear relationship with the risk of death (Figure 2). Specifically, the hazard ratios (HR) per 10 pg/mL increase were 1.05 (95% confidence interval (CI): 1.02–1.09; *p* = 0.002) for IL-6 and 1.03 (95% CI: 1.01–1.05; *p* < 0.001) for IL-10. In contrast, no significant associations were found in patients with CA125 ≤ 35 U/mL (HR = 0.99; 95% CI: 0.96–1.01; *p* = 0.317 for IL-6, and HR = 0.99; 95% CI: 0.97–1.01; *p* = 0.220 for IL-10).

Conversely to IL-6 and IL-10, the associations for IL-1β and TNF with mortality were not significantly influenced by CA125 levels (*p*-values for interactions = 0.490 and 0.960, respectively).

### 2.4. The Modifying Prognostic Role of Cytokine Network Across CA125: HF Readmissions

**Figure 3:**
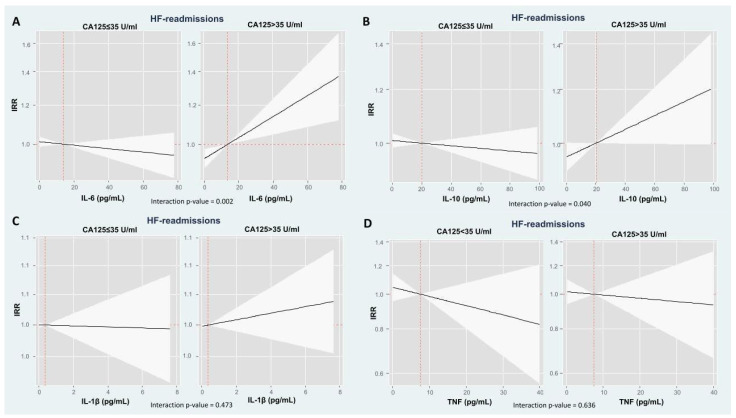
Risk of total heart failure admissions along the continuum of cytokines across CA125 status (CA125 ≤ 35 or >35 U/mL) in the multivariable model. Associations of IL-6 and IL-10 with HF readmission risk were modified by CA125 status, showing a positive relationship in patients with CA125 > 35 U/mL, but no significant association in those with CA125 ≤ 35 U/mL. IL-1β and TNF associations with HF readmissions were not affected by CA125 levels. Solid black lines represent IRR estimates, with shaded areas indicating 95% confidence intervals. The *p* for interaction tests whether the prognostic effect of cytokines on the risk of heart failure readmissions differs according to CA125 levels. (**A**) Interleukin-6: *p* for interaction = 0.002; (**B**) Interleukin-10: *p* for interaction = 0.040; (**C**) Interleukin-1β: *p* for interaction = 0.473; and (**D**) Tumor necrosis factor-α: *p* for interaction = 0.636. CA125: antigen carbohydrate 125, HF: heart failure; IL: interleukin, IRR: incidence rate ratio; TNF: tumor necrosis factor.

Similarly to the mortality endpoint, CA125 status modified the associations between IL-6 and IL-10 and the risk of total HF readmissions (*p*-values for interactions = 0.002 and 0.040, respectively). In patients with CA125 > 35 U/mL, IL-6 was significantly associated with a higher risk of HF rehospitalizations (Incidence rate ratios (IRR) = 1.05; 95% CI: 1.02–1.08; *p* = 0.002, per 10 pg/mL increase), while IL-10 showed a borderline positive association (IRR = 1.02; 95% CI: 1.00–1.05; *p* = 0.056) (Figure 3). On the contrary, in patients with CA125 ≤ 35 U/mL, neither IL-6 nor IL-10 were associated with HF readmission risk (IRR = 0.99; 95% CI: 0.98–1.01; *p* = 0.330, and IRR = 0.99; 95% CI: 0.98–1.01; *p* = 0.440, respectively). CA125 status did not modify the associations between IL-1β and TNF and the risk of HF readmissions (Figure 3).

## 3. Discussion

**Figure 4:**
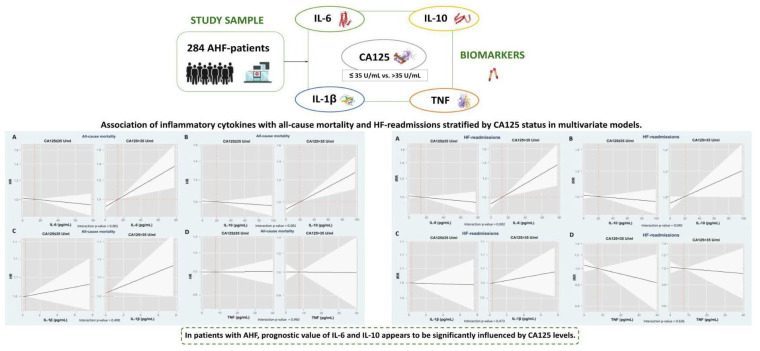
Summary of the design, results and conclusions of the study. Figures (A–D) show the results of risk of all-cause mortality and total heart failure admissions along the continuum of cytokines across CA125 status (CA125 ≤ 35 or >35 U/mL) in the multivariable model. Solid black lines represent HR and IRR estimates, with shaded areas indicating 95% confidence intervals. AHF: acute heart failure; CA125: carbohydrate antigen 125; HF: heart failure; IL: interleukin; TNF: tumor necrosis factor.

In this study, we found that the prognostic impact of systemic inflammatory cytokines, particularly IL-6 and IL-10, was significantly modified by circulating CA125 levels. Both cytokines showed an independent association with higher risk of all-cause mortality and total HF readmissions only among patients with increased CA125 concentrations (Figure 4). These findings are consistent with previous findings showing that the prognostic relevance of other fibrosis- and inflammation-related biomarkers—such as sST2 and Gal-3—appears to be influenced by CA125 status [20,21]. To our knowledge, this is the first study to show that CA125 modulates the prognostic role of inflammatory cytokines in acute HF, highlighting the convergence of two key pathophysiological axes, congestion and inflammation. These findings strengthen the emerging hypothesis that CA125 should not be regarded solely as a passive biomarker of fluid overload, but rather as an active biological modifier capable of amplifying inflammatory pathways and contributing to disease progression.

### 3.1. Prognostic Role of Inflammation in HF

Inflammation is a central pathophysiological mechanism in HF, contributing to disease progression through a complex interplay of cytokines, immune cells, and downstream molecular mediators [1,2]. Cytokines exert their effects in part through key intracellular signaling pathways, including MAPK and PI3K/Akt/mTOR, whose chronic activation regulates inflammation, apoptosis, and cell survival [22,23].

Persistent inflammatory activation promotes interstitial fibrosis, oxidative stress, endothelial dysfunction, and maladaptive cardiac remodeling, worsening congestion and multi-organ impairment [1,2]. Among pro-inflammatory pathways, the IL-1/IL-6/CRP axis has been most extensively studied. Experimental models have linked IL-1β and IL-6 to adverse ventricular remodeling and impaired cardiac function, while observational studies in clinical HF have reported associations between inflammatory cytokines, particularly IL-6, and worse functional status, higher congestion burden, renal dysfunction, anemia, and increased mortality risk [6,7,10,11,12,13,24,25]. Yet, prior data have been conflicting with respect to HF readmissions, and IL-6 has shown only modest additive value in some prognostic models [6,10].

IL-10, traditionally regarded as an anti-inflammatory cytokine, also exhibited a context-dependent association with prognosis. While generally considered protective, elevated IL-10 levels have been linked to increased cardiovascular risk in systemic inflammatory states [26], possibly indicating a maladaptive inflammatory milieu where compensatory anti-inflammatory responses are insufficient to counteract pro-inflammatory cytokine pathways.

As a novel finding, in the current study we only found such association (higher IL-6 and IL-10 with higher risk of adverse clinical outcomes) in patients with elevated CA125 levels, suggesting CA125 may be an active part of the inflammatory process in HF.

By contrast, we did not find that IL-1β and TNF were associated with long-term outcomes. Both cytokines are mechanistically involved in HF pathogenesis—IL-1β via inflammasome activation and myocardial remodeling [12], and TNF through apoptosis, oxidative stress, and contractile dysfunction [27,28]—but their prognostic role in contemporary populations remains uncertain. Prior studies have reported conflicting results, and therapeutic interventions targeting TNF have failed to improve outcomes [29]. Our findings reinforce the notion that the inflammatory network in HF is multifaceted and that prognostic implications may depend on the interplay of multiple mediators rather than isolated cytokine levels.

### 3.2. CA125 as a Potential Inflammatory Modulator in HF

CA125 is now an established biomarker in HF, with substantial prognostic value largely attributable to its strong correlation with signs of volume overload [15,30,31,32]. Recent evidence suggests that CA125 may exert additional mechanistic functions beyond being just a surrogate of congestion. CA125 is a large transmembrane mucin containing multiple potential cleavage sites [33]. Proteolytic processing releases its N-terminal fragment into the circulation, whereas the C-terminal domain (CTD) remains either membrane-bound or translocates to the nucleus, where it may function as a transcriptional co-regulator [34]. In malignancies such as ovarian cancer, nuclear translocation of the CTD activates invasion-related genes, thereby facilitating disease progression. The heavily glycosylated extracellular region of MUC16 enables protein–protein interactions that can activate intracellular signaling pathways. Evidence further indicates that the CA125 CTD can participate in an N-glycan–dependent complex with EGFR, β1 integrin, and Gal-3 on the cell surface [35,36]. These interactions promote epithelial-to-mesenchymal transition, a process implicated in cancer metastasis, organ fibrosis, and tissue remodeling, and are linked to soluble inflammatory mediators such as TNF, IL-6, and IL-1β [37]. This provides a plausible mechanistic framework through which inflammation and CA125 may converge to drive pathological effects in HF.

Epicardial adipose tissue is a relevant source of cytokines, including IL-6 and IL-1β, which contribute to fibroblast proliferation, collagen deposition, and myofibroblast activation—key processes in adverse cardiac remodeling [38]. Recently, MUC16 expression has been identified in epicardial adipose tissue from HF patients, where it correlates with pro-fibrotic and inflammatory markers, further reinforcing the biological link between CA125 expression to local myocardial inflammatory activity and extracellular matrix remodeling [19]. Moreover, experimental data suggest that CA125 can promote epithelial-to-mesenchymal transition (EMT), whereby mesothelial cells acquire a fibroblast-like phenotype, leading to excessive matrix deposition and fibrosis [39]. This mechanism may create a self-reinforcing cycle of inflammation, congestion, and structural damage. Alternatively, a simpler and pragmatic explanation is rooted in the extended half-life of CA125 (approximately 7–12 days) [15,16]. Within this framework, elevated CA125 levels, particularly when accompanied by a heightened cytokine milieu, may indicate persistent or chronic inflammatory activation rather than transient cytokine increase. This property underscores the potential of CA125 to capture cumulative inflammatory burden, thereby providing mechanistic insights beyond those afforded by rapidly fluctuating, short-lived inflammatory mediators.

In clinical research, prior studies have reinforced this concept by showing a significant interaction and potential modulatory effect of CA125 on other important inflammatory/fibrosis biomarkers. For example, the prognostic association of Gal-3 with adverse clinical outcomes in HF appeared dependent on CA125 levels, with deleterious effects observed only in patients with elevated CA125 values [20]. A similar interaction was reported with sST2, a biomarker representative of fibrotic and inflammatory processes in HF: in a subanalysis of the IMPROVE-HF trial, high sST2 levels were associated with a higher risk of cardiovascular and renal readmissions exclusively in patients with CA125 > 35 U/mL, but not in those with lower CA125 [21]. These findings suggest that CA125 could influence both the biological activity and the prognostic relevance of inflammatory pathways in HF. Our results extend this concept by directly evaluating systemic inflammatory cytokines, identifying a significant interaction for IL-6 and IL-10, whereby its association with long-term mortality and recurrent HF admissions was restricted to patients with CA125 > 35 U/mL.

### 3.3. Clinical Implications

From a clinical perspective, our findings suggest that CA125 may serve not only as a biomarker of congestion but also as a tool for refined risk stratification in patients with acute HF and heightened inflammatory burden.

Pro-inflammatory cytokines such as IL-6 and TNF impair endothelial barrier integrity, enhancing vascular permeability and promoting transudation of fluid into the interstitial space, thereby driving interstitial congestion and exacerbating volume overload [3,40]. Conversely, congestion itself serves as a potent trigger of inflammation. Experimental studies have shown that venous congestion can directly promote maladaptive inflammatory pathways [3,41]. Together, these mechanisms establish a self-reinforcing cycle: inflammation increases vascular leakage and fluid retention, intensifying congestion, while congestion further stimulates inflammatory signaling, leading to progressive extravascular fluid accumulation and interstitial expansion. Clinically, this phenotype manifests tissue inflammation and extravascular volume overload, which may remain undetected by conventional clinical evaluation or hemodynamic measurements [40,42,43].

The combination of elevated cytokines such as IL-6 and increased CA125 concentrations may identify a high-risk “congestive-inflammatory” phenotype, warranting a more comprehensive, multiparametric assessment of congestion and potentially differential therapeutic strategies. Conversely, cytokine elevation in the setting of low CA125 levels may reflect a transient or lower-grade secondary inflammatory response, with no prognostic implications.

Further studies are warranted to determine whether targeted anti-inflammatory therapies are of particular benefit in patients with elevated CA125 concentrations. This hypothesis is particularly relevant given ongoing trials, such as the HERMES trial (NCT05636176), which are evaluating IL-6 blockade in patients with HFpEF. Our findings may help guide patient selection for such therapies and inform the design of future biomarker-guided interventional studies.

### 3.4. Limitations

This study has several limitations. First, it was an observational study subject to potential biases. Second, although we adjusted for numerous clinical variables, residual confounding cannot be excluded. Third, biomarker measurements were performed at a single time point; thus, dynamic changes in cytokines or CA125 over time could not be assessed. Fourth, the study was conducted in a single center, which may limit the generalizability of our findings. Fifth, we did not assess biomarkers of oxidative stress, which may help disentangle the complex interplay between inflammation and congestion. Finally, with the current data we cannot unravel the exact biological meaning of the current associations.

## 4. Materials and Methods

### 4.1. Study Group and Protocol

We prospectively included a cohort of 284 patients admitted for acute HF in a tertiary hospital (Hospital Clínico Universitario de Valencia, Valencia, Spain) from 11 November 2010 to 1 August 2012. Patients with in-hospital death (*n*  = 13) and patients with a clinical diagnosis of infection on admission (*n*  =  21) were excluded. A comprehensive dataset of demographics, medical history, standard laboratory, echocardiographic parameters, and treatments at discharge was routinely recorded using pre-established registry questionnaires during the index hospitalization. Either patients with new-onset or worsening HF were enrolled in the registry. Acute HF was defined according to the running European Society of Cardiology Clinical Practice Guidelines though the study timeline. Treatment strategies were individualized following established guidelines operating when patients were included in the registry. The study was conformed to the principles outlined in the 1975 Declaration of Helsinki and was approved by the institutional, local review ethical committees. All patients gave informed consent.

### 4.2. Biomarker Measurements

Blood samples were collected within the first 24 h of admission. Plasma concentrations of CA125 were measured using immunoassay with the Elecsys CA125 II assay (Roche Diagnostics^®^, International, Rotkreuz, Switzerland), and IL-1β, IL-6, IL-10, and TNF-α were quantified with the multiplex immunoassay MILLIPLEX^®^ MAP (HSCYTMAG-60SK, Merck Millipore^®,^ Burlington, MA, USA), based on the Luminex^®^ xMAP^®^ technology, following the manufacturer’s instructions. CA125 was categorized according to the established cut-off of 35 U/mL. Cytokines were analyzed both as continuous variables and stratified by quartiles.

### 4.3. Follow-Up and Endpoints

The endpoints were all-cause mortality and total HF rehospitalizations during the entire follow-up. For the readmission endpoint, only unplanned readmissions were considered. The assessment of endpoints was performed by verifying the patient’s survival status or occurrence of readmission by reviewing electronic medical records of the public health care system, by paired investigators who were blinded to the biomarkers value. This assessment utilized data from the SIA-GAIA and Orion Clinics electronic databases, which comprehensively record all medical interactions occurring in the public health care system.

### 4.4. Statistical Analysis

Continuous variables are presented as mean ± standard deviation (SD) or median (P25 to P75), as appropriate. Discrete variables are expressed as percentages.

The association between biomarkers and all-cause mortality was evaluated using a Cox regression analysis, and results are reported as HR with 95% CI. For the readmission endpoint, negative binomial regression models were employed to simultaneously analyze the number of HF readmissions (as counts) and all-cause mortality (as a terminal event). Regression estimates for the readmission endpoint outcomes were mutually adjusted by means of shared frailty (accounting for the positive correlation between readmission and death). Risk estimates for HF-readmissions are expressed as IRR with 95% CI. Models for both endpoints were adjusted for clinically relevant prognosticators: age, sex, Charlson index, prior HF admissions, blood pressure, heart rate, hemoglobin, estimated glomerular filtration rate, NT-proBNP, LVEF, previous diuretic use, and HF medical therapies. Interaction terms were introduced to test whether associations between cytokines and outcomes varied according to CA125 status. The functional form of continuous variables, particularly the exposure of interest, was assessed using fractional polynomials, and appropriate transformations were applied where necessary to account for potential non-linear associations.

A 2-sided *p*-value of <0.05 was considered statistically significant for all analyses. All survival analyses were performed using STATA 17.1 (StataCorp. 2018. Stata Statistical Software: Release 14.1. College Station, TX, USA: StataCorp LP).

## 5. Conclusions

In patients with acute HF, the prognostic value of circulating inflammatory cytokines appears to be significantly influenced by CA125 levels. IL-6 and IL-10 were independently associated with long-term mortality and recurrent HF rehospitalizations only in those patients with elevated CA125 concentrations. These findings support the role of CA125 not only as a marker of congestion but also as a potential modulator of the inflammatory response in HF. Incorporating CA125 in the interpretation of inflammatory biomarkers may help identify high-risk subgroups and guide future personalized therapeutic strategies.

## Figures and Tables

**Table 1:** Baseline characteristics across CA125 status.

	Total Population (*n* = 284)	CA125 ≤ 35 U/mL (*n* = 90)	CA125 > 35 U/mL (*n* = 194)	*p*-Value
**Epidemiology and clinical findings**				
Age, years	72.8 ± 11.4	73.8 ± 10.1	72.3 ± 11.9	0.318
Women, *n* (%)	140 (49.3)	50 (55.6)	90 (46.4)	0.151
First HF admission, *n* (%)	212 (74.6)	64 (71.1)	148 (76.3)	0.351
Prior NYHA III/IV, *n* (%)	52 (18.3)	20 (22.2)	32 (16.5)	0.246
Hypertension, *n* (%)	221 (77.8)	75 (83.3)	146 (75.3)	0.128
Diabetes Mellitus, *n* (%)	132 (46.5)	34 (37.8)	98 (50.5)	0.045
Dyslipidemia, *n* (%)	143 (50.4)	49 (54.4)	94 (48.5)	0.347
Ischemic heart disease, *n* (%)	94 (33.1)	30 (33.3)	64 (33.0)	0.954
Valvular heart disease, *n* (%)	77 (27.1)	23 (25.6)	54 (27.8)	0.688
Heart rate, beats/min	97.7 ± 29.0	95.2 ± 26.9	96.0 ± 27.6	0.473
SBP, mmHg	150.7 ± 34.9	157.7 ± 36.4	147.5 ± 33.8	0.022
DBP, mmHg	82.1 ± 19.1	85.4 ± 19.9	80.5 ± 18.6	0.044
Peripheral edema, *n* (%)	200 (70.4)	53 (58.9)	147 (75.8)	0.044
Pleural effusion, *n* (%)	168 (59.2)	38 (42.4)	130 (67.0)	<0.001
**Circulating Biomarkers**				
Hemoglobin, g/dL	12.2 ± 1.9	12.4 ± 1.9	12.1 ± 1.9	0.154
NT-proBNP, pg/mL *	3540 (1894–7490)	2843 (1253–4775)	4043 (2064–8702)	0.006
Serum Creatinine, mg/dL	1.2 ± 0.6	1.2 ± 0.6	1.3 ± 0.6	0.500
GFR, mL/min/1.73 m^2^ *	63.6 ± 25.6	63.4 ±22.9	63.7 ± 26.8	0.906
IL-6, pg/mL *	13.8 (5.8–42.0)	17.5 (5.9–54.4)	13.6 (5.7–32.4)	0.025
IL-10, pg/mL *	20.2 (7.1–78.6)	20.2 (4.9–90.8)	20.4 (7.8–72.1)	0.551
IL-1β, pg/mL *	0.4 (0.1–2.4)	0.4 (0.1–2.5)	0.4 (0.1–2.2)	0.839
TNF, pg/mL *	7.6 (3.7–19.9)	7.5 (3.7–22.5)	7.6 (3.6–18.7)	0.527
CA125, U/mL *	69 (30–141)	19 (14–28)	111 (67–167)	<0.001
**Electrocardiography**				
AF, *n* (%)	127 (44.7)	41 (45.6)	86 (44.3)	0.874
**Echocardiography**				
LVEF ≥ 50%EF	157 ± 55.3	60 ± 66.7	97 ± 50	0.009
LAD, mm	42.6 ± 7.8	42.1 ± 6.3	42.9 ± 8.4	0.428
IVS, mm	11.3 ± 2.7	11.9 ± 2.9	11.1 ± 2.6	0.028
LV diastolic diameter, mm	54.8 ± 9.5	54.0 ± 9.3	55.2 ± 9.7	0.358
TAPSE, mm	18.5 ± 3.5	19.6 ± 3.6	17.9 ± 3.3	<0.001
**Treatment at discharge**				
Diuretics, *n* (%)	275 (96.8)	87 (96.7)	188 (96.9)	0.914
Beta-blockers, *n* (%)	205 (72.2)	59 (65.6)	146 (75.3)	0.090
RAAS inhibitors, *n* (%)	188 (66.2)	58 (64.4)	130 (67.0)	0.671
MRA, *n* (%)	41 (14.4)	7 (7.8)	34 (17.5)	0.030

Baseline characteristics of patients stratified by CA125 levels (≤35 U/mL vs. >35 U/mL). Continuous variables are shown as mean ± standard deviation or median (P25 to P75) *; categorical variables as *n* (%). *p*-values indicate differences between groups. HF, heart failure; NYHA, New York Heart Association; SBP, systolic blood pressure; DBP, diastolic blood pressure; GFR, glomerular filtration rate; IL, interleukin; TNF, tumor necrosis factor; AF, atrial fibrillation; LVEF, left ventricular ejection fraction; LAD, left atrial diameter; IVS, interventricular septum; TAPSE, tricuspid annular plane systolic excursion; RAAS, renin–angiotensin–aldosterone system; MRA, mineralocorticoid receptor antagonist.

**Table 2:** Rates of events per 100 patient-years across cytokine quartiles.

	All Cause Mortality HR (95% CI)	*p*-Value	HF Readmissions HR (95% CI)	*p*-Value
**IL-6**		0.245		0.989
Q1	17.9 (13.6–23.4)		27.5 (12.0–42.9)	
Q2	13.1 (0.98–17.4)		28.2 (13.6–42.6)	
Q3	18.0 (13.9–23.4)		30.9 (15.8–46.1)	
Q4	18.4 (14.1–23.9)		28.0 (19.0–46.2)	
**IL-10**		0.109		0.020
Q1	15.5 (11.7–20.1)		27.2 (10.9–43.5)	
Q2	13.1 (9.9–17.2)		14.8 (7.7–21.7)	
Q3	20.0 (15.4–25.9)		39.4 (20.8–57.9)	
Q4	19.0 (14.7–24.6)		36.1 (16.7–55.7)	
**IL-1β**		0.845		0.333
Q1	17.3 (13.2–22.8)		24.6 (13.3–35.8)	
Q2	17.8 (13.5–23.4)		42.0 (17.7–66.4)	
Q3	16.3 (12.4–21.6)		21.9 (11.3–31.8)	
Q4	15.4 (11.8–19.9)		29.0 (12.6–45.4)	
**TNF**		0.929		0.724
Q1	15.5 (11.9–20.5)		24.2 (10.4–37.9)	
Q2	16.9 (12.9–22.2)		35.3 (21.1–49.4)	
Q3	17.8 (13.7–23.2)		28.2 (13.7–42.6)	
Q4	16.3 (12.4–21.4)		26.8 (7.5–46.1)	

Hazard ratios with 95% confidence intervals for all-cause mortality and heart failure readmissions are shown according to quartiles (Q1–Q4) of interleukin-6, interleukin-10, interleukin-1β, and tumor necrosis factor. Global *p*-values correspond to the overall comparison among quartiles for each cytokine. CI: confidence interval; HF: heart failure; HR: hazard ratio; IL: interleukin, TNF: tumor necrosis factor.

**Table 3:** Cytokines and risk of adverse outcomes in multivariable models in the whole sample.

	IRR (95% CI)	*p*-Value
**All-cause mortality**		
IL-6, per increase in 10 pg/mL	1.01 (0.99–1.02)	0.637
IL-10, per increase in 10 pg/mL	1.00 (0.99–1.01)	0.721
IL-1β, per increase in 10 pg/mL	1.06 (0.99–1.14)	0.100
TNF, per increase in 10 pg/mL	1.00 (0.97–1.04)	0.971
**Total HF-readmissions**		
IL-6, per increase in 10 pg/mL	1.01 (0.99–1.02)	0.752
IL-10, per increase in 10 pg/mL	1.00 (0.99–1.01)	0.967
IL-1β, per increase in 10 pg/mL	1.02 (0.94–1.11)	0.651
TNF, per increase in 10 pg/mL	0.962 (0.90–1.03)	0.250

Incidence rate ratios with 95% confidence intervals and *p*-values are presented for interleukin-6, interleukin-10, interleukin-1β, and tumor necrosis factor, expressed per 10 pg/mL increase in circulating levels. CI: confidence interval; HF: heart failure; IL: interleukin, IRR: incidence rate ratio; TNF: tumor necrosis factor.

## Data Availability

Data is available through a request directed to the corresponding author upon reasonable request.

## Abbreviations

HF	Heart failure
CA125	Carbohydrate Antigen 125
IL-6	Interleukin 6
IL-10	Interleukin 10
IL-1β	Interleukin 1β
TNF	Tumor necrosis factor
MUC16	Mucin 16
Gal-3	Galectin-3
sST2	Soluble ST2
SD	Standard deviation
HR	Hazard ratio
CI	Confidence interval
IRR	Incidence rate ratios
LVEF	Left ventricular ejection fraction
CTD	C-terminal domain
EMT	Epithelial-to-mesenchymal transition

## Appendix A

**Table A1:** Supplementary table of baseline characteristics across CA125 status.

	Total Population (*n* = 284)	CA125 ≤ 35 U/mL (*n* = 90)	CA125 > 35 U/mL (*n* = 194)	*p*-Value
**Epidemiology and clinical findings**				
Current smoker, *n* (%)	24 (8.5)	6 (6.7)	18 (9.3)	0.462
PAD, *n* (%)	19 (6.7)	6 (6.7)	13 (6.7)	0.991
Charlson index	2.2 ± 1.9	2.0 ± 1.7	2.3 ± 1.9	0.267
**Circulating Biomarkers**				
Hematocrit (%)	37.5 ± 5.4	38.1 ± 5.5	37.2 ± 5.4	0.205
WBC count, 10^9^/L	9.4 ± 3.7	10.6 ± 4.0	8.9 ± 3.4	<0.001
Neutrophiles, 10^9^/L	6.9 ± 2.9	7.7 ± 3.1	6.5 ± 2.7	0.002
Iron, µg/dL	49.6 ± 15.9	54.7 ± 14.4	47.3 ± 16	<0.001
**Electrocardiography**				
QRS > 120 ms, *n* (%)	96 (33.8)	31 (34.4)	65 (33.5)	0.876
